# 

**Published:** 1875-10

**Authors:** 


*The American Academy of Dental Surgery of New York.*—A special meeting will be held at the College of Physicians and Surgeons, New York city, on Tuesday and Wednesday, October 19th and 20th, 1875, at 10-30 o'clock A. M. Also, the regular meeting at 50 West 35th Street, on Tuesday, the 19th of Oct. at 7 1-2 o'clocg, P. M. The order of exercises consists of Prayer, by Rev. Hugh Miller Thompson, D. D , Rector of Christ Church, N. Y. Greetings by Mayor Wickham, and the President, Dr. Perine. An address on behalf of the medical profession on the " Relations of Dental Science to Medicine," by Prof. Wm. H. Hammond, M. D., of the University of New York. An address on behalf of the dental profession, by W. C. Barrett, D. D. S., of Buffalo, N. Y. Essays by Drs. Jas. E. Garretson, Alfred L. Carroll, A. P. Merrill, C S. Hurlbut, J. B. Wilmott, and W. T. Elliott, to be followed by discussions.

The Committee of Arrangements consist of Drs. A. P. Merrill, F. S. Howard and John P. Adams.

### BIBLIOGRAPHICAL.

### TO READERS AND CORRESPONDENTS.

Communications intended for publication, and exchange journals and papers, should be addressed to the EDITOR OF THE AMERICAN JOURNAL OF DENTAL SCIENCE, 259 N. Eutaw St., Baltimore, Md. All letters relating to the business of the journal, or containing cash remittances, to be directed to Messrs. SNOWDEN & COWMAN. Articles for publication should be received by the editor at least three weeks previous to the first day of the month on which the number in which it is designed for them to appear, is issued.

### CONTENTS:

### ORIGINAL COMMUNICATIONS.

ARTICLE I.—Probabilities. By Dr. G. V. Black.................. 241
ARTICLE II.—Mechanical Dentistry. By Dr. O. Willson.................. 250

### SELECTED ARTICLES.

### MONTHLY SUMMARY.

Dispensing with Rain.................. 287
Ether vs. Chloroform.................. 288
Effects of the Inhalation of Chloroform during Parturition, on the Fœtus.................. 288

☞The American Journal of Dental Science will contain fifty pages of reading matter in each number, making a volume of not less than

*Six Hundred* pages

EXCLUSIVE OF ADVERTISEMENTS.

THE

### AMERICAN JOURNAL

OF

### DENTAL SCIENCE.

F. J. S. GORGAS, M. D., D. D. S., Editor.

The *Journal* is issued on the first of each month and contains not less than *fifty* pages of reading matter in each number, exclusive of advertisements, making a volume of *six hundred* pages per annum.

In each number will be found Original Communications, Correspondence, Selected Articles, Monthly Summary, Bibliographical Notices and Editorial Matter, and every effort will be exerted to render the *Journal* worthy of the patronage of the Dental profession.

A generous co-operation is respectfully solicited from the members of the profession; and as the *Journal* has now a printing office of its own, which materially lessens the cost of its publication, it will be issued at the reduced subscription price of

**$2.50 Per Annum in Advance.**

Communications are respectfully solicited on all subjects pertaining to the practice of dentistry, as well as reports of cases occurring in dental practice.

### RATES OF ADVERTISING.

| | 1 MO. | 2 MO. | 3 MO. | 6 MO. | 12 MO. |
|---|---|---|---|---|---|
| 1 PAGE. | $15 00 | $22 50 | $30 00 | $50 00 | $100 00 |
| ½ " | 10 00 | 15 00 | 20 00 | 30 00 | 50 00 |
| ¼ " | 7 00 | 11 00 | 15 00 | 20 00 | 30 00 |
| ⅛ " | 5 00 | 8 00 | 11 00 | 15 00 | 20 00 |

All original communications designed for publication, works for review, new instruments for notice, exchanges, &c., should be directed to the editor, 259 N. Eutaw St., Baltimore, Md.

All advertisements and subscriptions should be addressed to

SNOWDEN & COWMAN,

*Publishers of the Am. Journal of Dental Science.*

86 W. FAYETTE ST. BET. CHARLES & LIBERTY STS.,

BALTIMORE, MD.

